# Comparative Genomic Analysis Reveals the Potential Risk of *Vibrio parahaemolyticus* Isolated From Ready-To-Eat Foods in China

**DOI:** 10.3389/fmicb.2019.00186

**Published:** 2019-02-07

**Authors:** Rui Pang, Tengfei Xie, Qingping Wu, Yanping Li, Tao Lei, Jumei Zhang, Yu Ding, Juan Wang, Liang Xue, Moutong Chen, Xianhu Wei, Youxiong Zhang, Shuhong Zhang, Xiaojuan Yang

**Affiliations:** ^1^State Key Laboratory of Applied Microbiology Southern China, Guangdong Provincial Key Laboratory of Microbial Culture Collection and Application, Guangdong Open Laboratory of Applied Microbiology, Guangdong Institute of Microbiology, Guangzhou, China; ^2^Department of Food Science and Technology, Jinan University, Guangzhou, China; ^3^College of Food Science, South China Agricultural University, Guangzhou, China

**Keywords:** *Vibrio parahaemolyticus*, ready-to-eat foods, genomics, potential risk, biofilm

## Abstract

*Vibrio parahaemolyticus* is a major foodborne pathogen associated with the consumption of aquatic products. The presence of this bacterium in ready-to-eat (RTE) foods has recently been reported. However, the genomic features and potential risks of *V. parahaemolyticus* isolated from RTE foods are poorly understood. To help understand the genome-wide characteristics of RTE food isolates, the complete genomes of 27 RTE food isolates were sequenced and compared to those of 20 clinical and 19 other environmental (e.g., water and aquatic product source) isolates using a comparative genomics approach. Analysis revealed that *V. parahaemolyticus* RTE food isolates had higher numbers of genes on average and possessed more accessory genes than isolates from other sources. Most RTE food isolates were positive for some known virulence-associated genes and pathogenicity islands (PAIs), and some of these isolates were genetically homologous to clinical isolates. Genome-wide association analysis revealed 79 accessory genes and 78 missense single-nucleotide polymorphisms that affected 11 protein-coding genes were significantly associated with RTE food sources. These genes were mostly involved in defense mechanisms and energy production and conversion according to functional annotation in the COG database. KEGG Pathway analysis showed that these genes mainly affected the biofilm formation of *V. parahaemolyticus*, and subsequent experiments confirmed that nearly all RTE food isolates possessed the ability to form biofilm. The biofilm formation can facilitate the persistence of *V. parahaemolyticus* in RTE foods, and the presence of virulence-associated genes poses a pathogenic potential to humans. Our findings highlight the potential risk of *V. parahaemolyticus* in Chinese RTE foods and illustrate the genomic basis for the persistence of these isolates. This study will aid in re-evaluating the food safety threats conferred by this bacterium.

## Introduction

*Vibrio parahaemolyticus* is a gram-negative, halophilic bacterium that is commonly found in estuarine and marine environments worldwide. This microorganism is recognized as one of the most prevalent foodborne pathogens and typically causes acute gastroenteritis in humans (Letchumanan et al., 2014). This bacterium grows preferentially in warm and low-salinity marine water and sometimes colonizes aquatic hosts such as mollusks, shrimp, and fish (Depaola et al., 1990). Due to its frequent presence in aquatic products, *V. parahaemolyticus* infections are commonly associated with the consumption of raw or undercooked seafood (Ceccarelli et al., 2013). However, our recent report demonstrated the presence of this bacterium in Chinese ready-to-eat (RTE) foods (Xie et al., 2016), a specific type of source that was rarely associated with *V. parahaemolyticus* infections previously.

RTE foods, such as deli meat, roasted poultry, and cold vegetable dishes, are very popular in China because of their taste and convenience. Unlike in other types of food, no heat processing is needed for RTE foods before consumption. Therefore, these foods tend to be implicated in foodborne illnesses more than other types of food that must be cooked before eating (Tian et al., 2008). Previous studies have shown that RTE foods available in Chinese markets are contaminated by foodborne pathogens such as *Listeria monocytogenes* (Chen et al., 2014; Wu et al., 2015), *Staphylococcus aureus* (Yang et al., 2016a), *Salmonella* spp. (Yang et al., 2016b), and *Cronobacter* spp. (Xu et al., 2015). The contamination rate of *V. parahaemolyticus* in Chinese RTE foods can reach 7.63% (Xie et al., 2016). While most food industry processes in China include critical disinfection techniques, contamination with pathogens still occasionally occurs. One of the major reasons for this is that many bacteria possess the ability to form biofilms (Sun and Dong, 2009). By adhering to food surfaces and forming biofilms, bacteria may become a persistent source of contamination, threatening the microbiological quality and safety of food products and perhaps even resulting in foodborne disease and economic losses (Van Houdt and Michiels, 2010). However, there are no reports describing the biofilm formation ability of RTE food-isolated pathogens. In consideration of the mass sale of these foods in China, evaluating the potential pathogenicity of microbes in RTE foods is of critical importance for food security.

Bacterial pathogenicity is usually associated with the presence of virulence factors. The pathogenicity of *V. parahaemolyticus* is mainly attributed to the production of two major virulence factors: thermo-stable direct hemolysin (TDH), encoded by the *tdh* gene, and TDH-related hemolysin, encoded by the *trh* gene (Honda, 1993). TDH has hemolytic activity on Wagatsuma agar, designated the Kanagawa phenomenon (KP), and is involved in cytotoxicity (Miyamoto et al., 1969), while TRH is considered to have a similar action (Honda et al., 1988). The presence of multiple pathogenicity islands (PAIs) is also considered a feature of pathogenic *V. parahaemolyticus*. For example, the *tdh* gene is located in VPAI-7 (*tdh-*PAI), while the *trh* gene is located in the *trh-*PAI (Chen et al., 2011). In addition to the *tdh-*PAI, pandemic *V. parahaemolyticus* possesses six additional PAIs, VPAI-1 to VPAI-6 (Hurley et al., 2006). All of these PAIs are predominantly present among pandemic isolates and may have been acquired from other *Vibrio* species (VPAI-1 to VPAI-3) or *Shewanella* species (VPAI-5 and VPAI-6) by horizontal gene transmission (HGT). Moreover, comparative genomic analyses have revealed that pathogenic *V. parahaemolyticus* encodes two type III secretion systems (T3SS), while environmental isolates commonly encode only a single system (T3SS1) (Makino et al., 2003). T3SS1 contributes to the cytotoxicity of *V. parahaemolyticus* but does not appear to play a significant role in intestinal colonization or the induction of intestinal pathology (Park et al., 2004). In contrast, T3SS2 is essential for intestinal colonization and is derived from two separate lineages, one found on VPAI-7 with the *tdh* gene (T3SS2α), and the other found with the *trh* gene (T3SS2β) (Okada et al., 2009; Broberg et al., 2011). Similar to T3SS, *V. parahaemolyticus* also have two type VI secretion systems (T6SS). T6SS2 is found in all strains, while T6SS1 is mostly associated with pathogenic isolates and may contribute to virulence (Salomon et al., 2014).

Our previous studies found that none of the isolates from RTE foods carried the *tdh* or *trh* genes (Xie et al., 2016). However, further testing to detect the presence of other virulence factors is lacking, and the risk of *V. parahaemolyticus* in RTE foods still remains uncertain. Although molecular subtyping by enterobacterial repetitive intergenic consensus sequence PCR (ERIC-PCR) typing and multilocus sequence typing (MLST) has revealed the genetic diversity of *V. parahaemolyticus* isolates from RTE foods (Xie et al., 2016), the genetic relationship between these isolates and pathogenic isolates remains unknown owing to the lack of genome-wide information on *V. parahaemolyticus* RTE food isolates. Therefore, the aim of this study was to assess the risk of *V. parahaemolyticus* in RTE foods through a whole-genome sequencing strategy. We present a comparative genomic analysis of multiple isolates from RTE foods and clinical and other environmental sources (e.g., environmental water and aquatic products). Pan-genome analysis revealed that *V. parahaemolyticus* RTE food isolates possessed more accessory genes than isolates from other sources on average. At the same time, some RTE food isolates were found to carry several known virulence-associated genes. We also identified multiple genes and single-nucleotide polymorphisms (SNPs) that were closely correlated to RTE food sources, and these factors may contribute to defense processes and biofilm formation in *V. parahaemolyticus*. The results of this study provide critical insights into the genomic features of *V. parahaemolyticus* isolated from RTE foods and may aid in improving strategies for microbiological risk assessment.

## Materials and Methods

### Bacterial Strains

Twenty-seven isolates of *V. parahaemolyticus* were collected from different cities and RTE foods in China (Table S1). Bacteria were grown overnight in 3% NaCl trypticase soy broth (TSB) before genomic DNA extraction. We also selected 20 clinical and 19 other environmental isolates for which genome sequences were available from the NCBI database (Table S1). All analyzed isolates were collected in Asia, and their serotypes, sources, and years of collection are listed in Table S1.

### Genome Sequencing and Assembly

Genomic DNA was obtained from *V. parahaemolyticus* isolates by lysing the bacteria with proteinase K followed by DNA extraction and purification with the Ezup Column Bacteria Genomic DNA Purification Kit (Sangon, Shanghai, China) according to the manufacturer's protocol. Each DNA sample was then fragmented into 400-bp fragments by a Covaris M200 sonicator and used to generate sequencing libraries. Whole genomes were sequenced with the Life Ion S5 platform to an average coverage of 100×. Clean reads were used for *de novo* assembly with SPAdes v3.6.2 (Bankevich et al., 2012).

### Pan-Genome Analysis

Genome annotation was performed on all analyzed isolates using Prokka v1.11 (Seemann, 2014). The output of Prokka was used to construct the pan-genome using Roary v3.11.2 (Page et al., 2015). A core genome was determined for each isolate using a 99% cutoff, with a BLASTP identity cutoff of 85%. To identify accessory genes overrepresented in RTE food isolates, we used Scoary (Brynildsrud et al., 2016). For this analysis, we used the isolate source as the trait of interest, and we adjusted the *P-*values for multiple comparisons using the Benjamini and Hochberg method.

### SNP Calling and Genome-Wide Association Analysis

Whole-genome alignments of all strains were constructed with Parsnp v1.2 (Treangen et al., 2014) using the RIMD2210633 genome as a reference, and PhiPack filtering (Bruen et al., 2006) was enabled to remove SNPs located in regions of recombination. SNP sites were then extracted by Harvesttools (Treangen et al., 2014) and annotated with SnpEff (Cingolani et al., 2012). We also used Gubbins (Croucher et al., 2015) to conduct recombination analysis on the core genome alignments generated by Harvesttools.

The core genome SNP alignment was used to estimate the genetic population structure using the hierBAPS module of the BAPS software program, which fits lineages to genome data using nested clustering (Cheng et al., 2013). The estimation used three independent interactions with 15, 30, and 45 clusters at levels 1–3 of the hierarchy, respectively.

To test for evidence of RTE food-associated SNP variation, we used the Cochran–Mantel–Haenszel (CMH) test as implemented in PLINK (Purcell et al., 2007). To account for the population structure, we used the BAPS level 3 clustering in the CMH test. Only SNPs with a minor allele frequency (MAF) > 0.01 across all isolates were used for association analysis. An association was considered statistically significant if the adjusted *P*-value (Bonferroni-corrected) of the SNP was less than 0.05. To enable that the SNPs were specifically associated with the RTE food isolates, we filtered out the non-significant SNPs from the above outliers in the comparisons of RTE food isolates to clinical and other environmental isolates separately (the Fisher's Exact test, *P*-value ≥ 0.05).

### Phylogenetic Analysis

Based on the SNP alignment, a maximum-likelihood (ML) phylogenetic tree was constructed using FastTree v. 2.1.10 with the general time-reversible (GTR) and gamma model of nucleotide substitution (Price et al., 2010). The ML phylogeny was visualized and annotated using iTOL (Letunic and Bork, 2016).

### Functional Analysis

To assess associations between RTE food-related accessory genes or missense SNP-containing genes and functional gene categories, we used BLAST to compare representative gene sequences with the NCBI Non-redundant (NR) and Clusters of Orthologous Groups (COG) protein database. Pathway annotation was conducted using the Kyoto Encyclopedia of Genes and Genomes (KEGG) Automatic Annotation Server (KAAS).

### Biofilm Formation of *V. parahaemolyticus* Isolates

A crystal violet staining method was applied to examine the biofilm-forming abilities of *V. parahaemolyticus* RTE food isolates, as described by Ye et al. (2014). Briefly, the isolates were inoculated into 5 mL TSB and grown at 37°C with shaking at 150 rpm for 14 h. Thirty microliters of cultures with an optical density at 590 nm (OD_590_) of 0.65 were inoculated into 96-well polystyrene plates containing 90 μL fresh TSB and incubated at 37°C for 24 h. The plates were rinsed three times with deionized water, and adherent bacterial cells were stained with 1% crystal violet for 30 min. After rinsing three times with deionized water, the crystal violet was liberated by acetic acid (30%). The OD_590_ values of each well were measured. Each strain was assessed a minimum of three times. The OD values of the tested samples were normalized to that of the negative control (OD_c_), and biofilm formation ability was determined according to a previous metric: strong biofilm (OD > 4OD_c_), intermediate biofilm (2OD_c_ < OD < 4OD_c_), weak biofilm (OD_c_ < OD < 2OD_c_), and no biofilm (OD < OD_c_) (Ding et al., 2014). The formed biofilms were observed under a scanning electron microscope (S-3000N, Hitachi, Tokyo, Japan).

### Data Accessibility

The draft genome sequences of *V. parahaemolyticus* generated in this study are submitted to the NCBI database under BioProject PRJNA491373. The accession numbers were QYTA00000000, QYSZ00000000, QYSY00000000, QYSX00000000, QYSW00000000, QYSV00000000, QYSU00000000, QYST00000000, QYSS00000000, QYSR00000000, QYSQ00000000, QYSP00000000, QYSO00000000, QYSN00000000, QYSM00000000, QYSL00000000, QYSK00000000, QYSJ00000000, QYSI00000000, and QYSH00000000.

## Results

### General Genomic Features of *V. parahaemolyticus* RTE Food Isolates

We sequenced 27 isolates of *V. parahaemolyticus* collected from RTE foods sourced from different regions of China. As a comparison, we combined these data with 39 genome sequences of *V. parahaemolyticus* isolated from clinical and other environmental sources (Table S1). To exclude geographical influence, only isolates sampled from Asia were selected.

**Figure 1:**
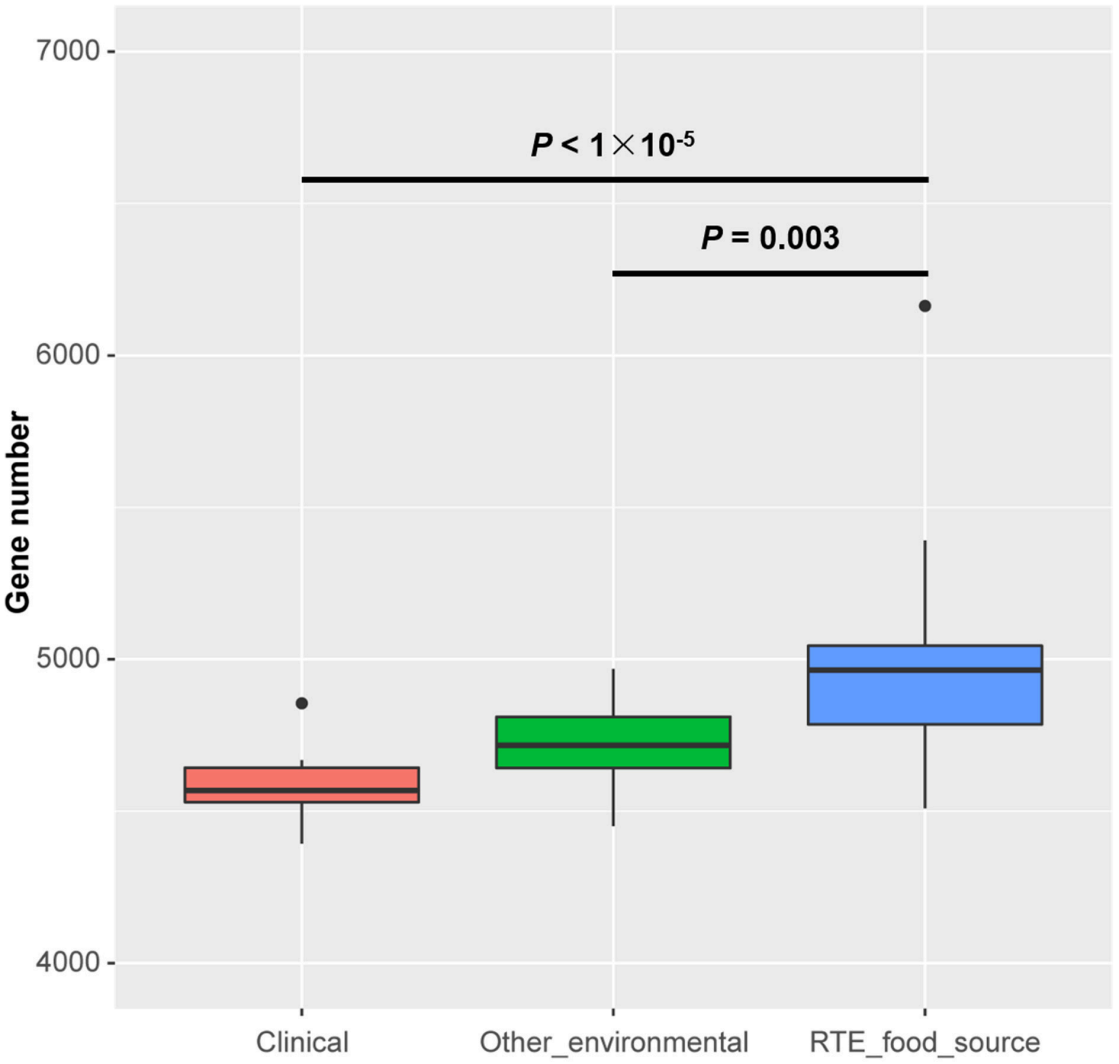
Genome size differences in *Vibrio parahaemolyticus* isolates from RTE foods and other sources. Boxes show the medians and upper and lower quartiles; whiskers show the most extreme values within 1.5 times the interquartile range. Clinical: *n* = 20; Other environmental: *n* = 19; RTE food sources: *n* = 27. *P*-values are obtained according to the one-way analysis of variance.

The size of the draft genomes of the 27 RTE food isolates (Table S1) ranged from 4.95 Mb (Vp34) to 5.99 Mb (Vp26). These isolates contained an average of 4,952 genes, which was significantly more genes than among other environmental (4,718 on average) or clinical isolates (4,580 on average) (Figure 1). This observation suggested that the persistence of *V. parahaemolyticus* in RTE foods depended on an increased abundance of accessory genes.

**Figure 2:**
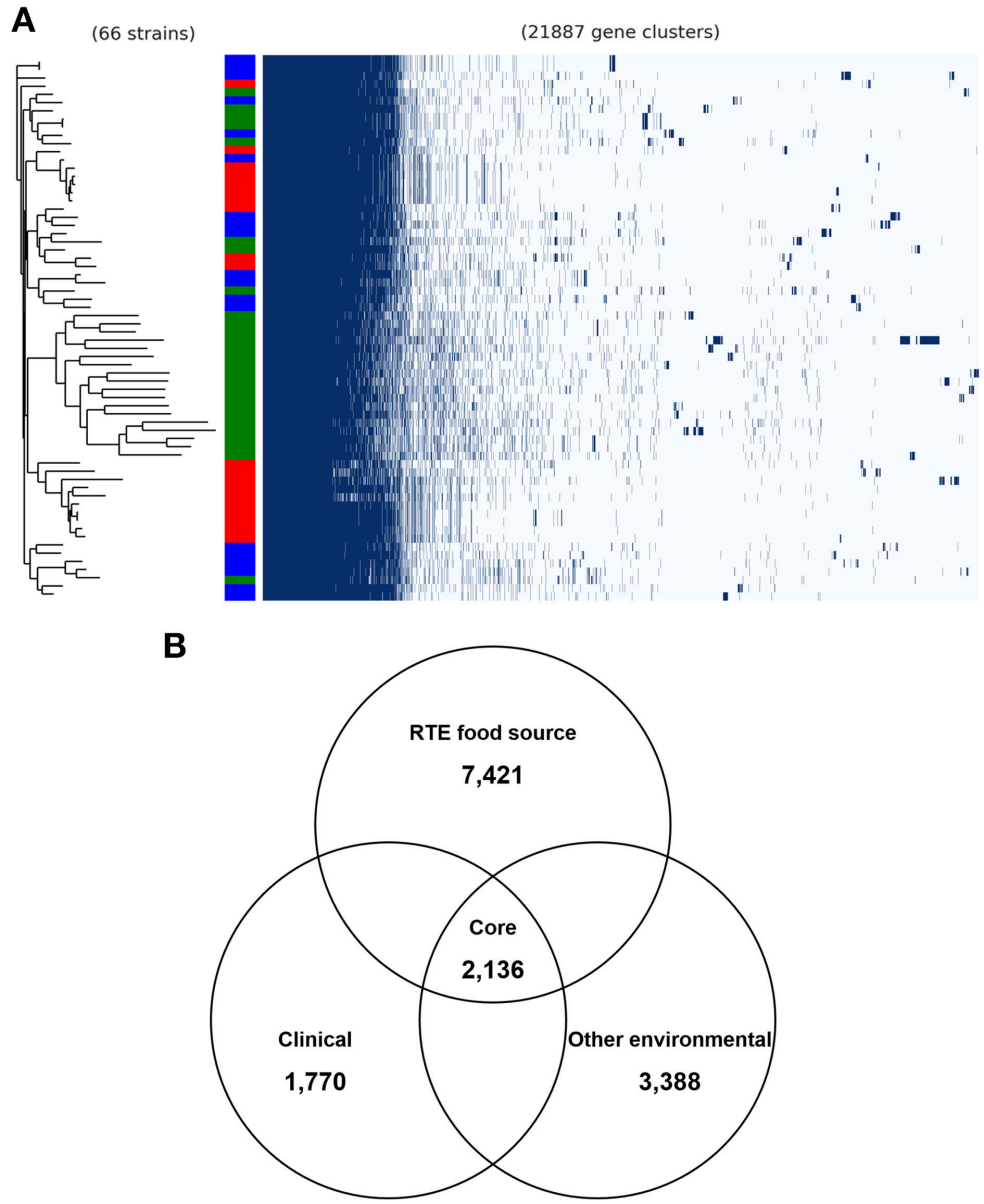
General genomic features of *V. parahaemolyticus* isolates. **(A)** the pangenome distribution of *V. parahaemolyticus* isolates that was constructed from the genome sequences of 66 strains. Green in color bar indicates RTE food isolates, while red indicates clinical isolates and blue indicates other environmental isolates. **(B)** comparison of the unique accessory genes of RTE food-sourced, clinical, and other environmental isolates.

To validate the above inference, we analyzed the pangenome of all isolates. This revealed a pangenome consisting of 21,887 protein-coding genes (Figure 2A). Notably, most RTE food isolates (66.7% in the same clade) shared a similar pattern of accessory gene presence and absence, revealing the existence of potential gene clusters that are needed by *V. parahaemolyticus* to persist in RTE foods. Within the pangenome, 2,136 genes were present in all genomes (core genes) (Figure 2B), occupying 35–49% of each isolate's genome. A total of 7,421 accessory genes unique to RTE foods were identified, representing a much higher number than the number of genes specific to clinical and other environmental isolates (1,770 and 3,388 accessory genes respectively). This finding reinforced the above result that more accessory genes are presented in RTE foods isolates.

Therefore, we performed a pangenome-wide association analysis to identify accessory genes that are overrepresented in RTE food isolates using Scoary. We found that 109 genes were significantly associated with the RTE food trait, with 79 genes overrepresented in RTE food isolates (Table S2). The number of overrepresented genes was obviously smaller that the number of accessory genes unique to RTE foods. The reason was that most of those unique accessory genes were only presented in only one or two RTE food isolates. Instead, most of these overrepresented genes were present in over half of the RTE food isolates but were rarely present in other isolates. This reveals the potential key role of these genes in the persistence of *V. parahaemolyticus* in RTE foods.

We also determined the presence of known *V. parahaemolyticus* virulence-associated genes in all RTE food-sourced genomes using pangenome analysis. As reported by Xie et al. (2016), all RTE food isolates were *tdh*-negative (Table 1). Similarly, none of these isolates carried the *trh* gene except for one isolate, Vp19. This isolate also possessed most genes belonging to T3SSβ, as well as the complete complement of T6SS1 and T6SS2 genes. Genes belonging to T3SS2α and VPAI-5 to VPAI-7 were also absent from all RTE food-sourced genomes. However, all of these isolates carried complete or partial complements of T6SS2 genes, and over half of them carried T6SS1. In addition, an incomplete VPAI-4 was present in 26% of RTE food isolates, and several isolates showed the presence of VPAI-1, VPAI-2, or the filamentous vibriophage f237. These results highlighted the pathogenic potential of some *V. parahaemolyticus* RTE food isolates.

**Table 1:** Detection of virulence-associated genes in the sequenced genomes of *V. parahaemolyticus* RTE foods isolates.

**Stranis**	**serotype**	**tdh**	**trh**	**T3SS2α**	**T3SSβ**	**VPAI-1**	**VPAI-2**	**VPAI-3**	**VPAI-4**	**VPAI-5**	**VPAI-6**	**VPAI-7**	**T6SS1**	**T6SS2**	**f237**
V146	O1:KUT	–	–	–	–	–	–	–	±	–	–	–	–	+	±
V147	O6:KUT	–	–	–	–	–	–	–	–	–	–	–	±	+	–
V148	Unknown	–	–	–	–	–	±	–	±	–	–	–	–	±	–
V187	O2:KUT	–	–	–	–	–	–	–	–	–	–	–	–	±	–
V209	O2:KUT	–	–	–	–	–	–	–	–	–	–	–	–	+	–
Vp10	O4:KUT	–	–	–	–	–	–	–	–	–	–	–	±	±	–
Vp12	O4:KUT	–	–	–	–	–	–	–	–	–	–	–	–	±	–
Vp19	O4:KUT	–	+	–	±	–	–	–	–	–	–	–	+	+	–
Vp2	O2:KUT	–	–	–	–	–	–	–	–	–	–	–	–	+	–
Vp22	O2:KUT	–	–	–	–	–	+	–	–	–	–	–	–	+	–
Vp23	O6:KUT	–	–	–	–	±	–	–	–	–	–	–	+	+	–
Vp26	O4:KUT	–	–	–	–	–	+	–	±	–	–	–	±	+	–
Vp27	O7:KUT	–	–	–	–	–	–	–	±	–	–	–	±	+	–
Vp28	O2:KUT	–	–	–	–	–	–	–	–	–	–	–	±	±	–
Vp3	O12:KUT	–	–	–	–	–	–	–	±	–	–	–	±	±	±
Vp30	O5:KUT	–	–	–	–	–	–	–	–	–	–	–	–	+	–
Vp34	O2:KUT	–	–	–	–	–	–	–	–	–	–	–	–	+	–
Vp37	O5:KUT	–	–	–	–	–	–	–	–	–	–	–	±	+	–
Vp38	O1:KUT	–	–	–	–	–	–	–	–	–	–	–	±	+	–
Vp40	O8:KUT	–	–	–	–	–	–	–	±	–	–	–	±	+	–
Vp41	O1:KUT	–	–	–	–	–	–	–	±	–	–	–	±	+	–
Vp43	O2:KUT	–	–	–	–	–	–	–	–	–	–	–	–	+	–
Vp45	O2:KUT	–	–	–	–	–	–	–	–	–	–	–	–	±	–
Vp46	O4:KUT	–	–	–	–	–	–	–	–	–	–	–	±	+	–
Vp47	O3:KUT	–	–	–	–	–	–	–	–	–	–	–	±	±	±
Vp5	O12:KUT	–	–	–	–	–	–	–	–	–	–	–	–	+	–
Vp9	O4:KUT	–	–	–	–	–	–	–	–	–	–	–	±	+	–

*+Indicates a gene or more than 80% genes within a region were detected, ± indicates that more than half and less than 80% of the genes within a region were detected*.

### Phylogenetic Structure of *V. parahaemolyticus* RTE Food Isolates

**Figure 3:**
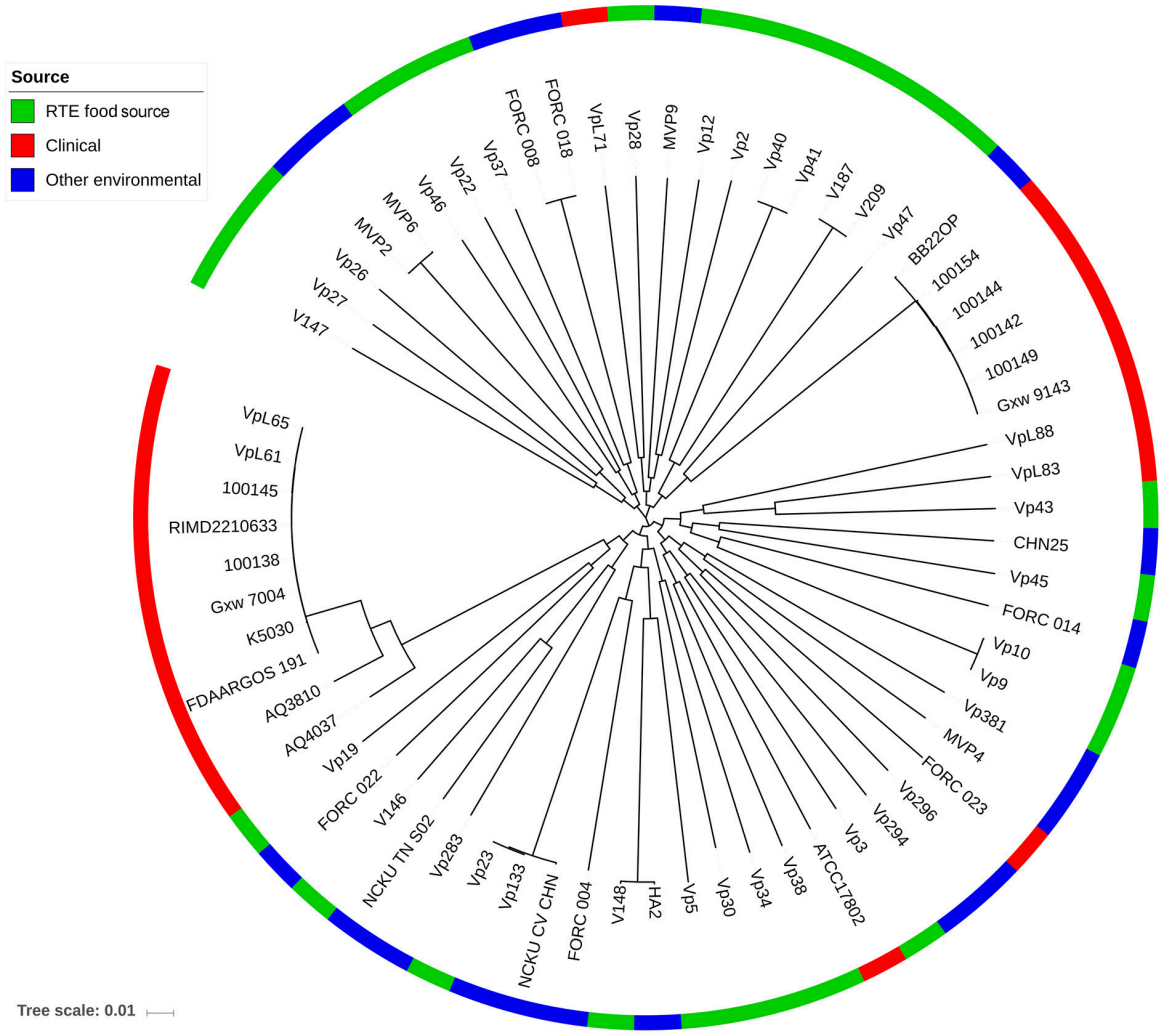
Maximum likelihood phylogeny of 68,410 non-recombining SNP variants from the core genome of 66 *V. parahaemolyticus* isolates. Isolates from different sources are indicated with different colors.

To provide insight into the genetic diversity of *V. parahaemolyticus* RTE food isolates, an ML phylogenetic tree was constructed using 68,410 non-recombining core genome SNPs (Figure 3). The clinical isolates used in our study were divided into two major lineages: one corresponding to the O3:K6 serotype pandemic strain (Nair et al., 2007), and the other containing two sub-clades was closed to the pathogenic environmental isolate BB22OP (Jensen et al., 2013). Most of the RTE food-sourced and environmental isolates were genetically distinct from these two pathogenic lineages. However, isolate Vp19 was genetically homologous to AQ4037, a pre-pandemic O3:K6 isolate that also possessed the ability to cause foodborne disease (Chen et al., 2011). AQ4037 possessed the same virulence-associated genes as Vp19 (*trh*, T3SSβ, T6SS1, and T6SS2), revealing that RTE food-sourced and clinical *V. parahaemolyticus* could share a similar genetic architecture. A similar inference was also obtained from isolate Vp43, which was genetically homologous to VpL83, a clinical *tdh*- and *trh*-negative isolate that may possess other uncharacterized virulence factors.

### Identification of RTE Food-Related SNPs

To clarify whether any SNP variants were consistently associated with RTE food isolates, we used the software program PLINK to analyze SNPs in the core genome. Before association analysis, we used BAPS to infer the population structure. Under the threshold of 30 clusters at the third level of the hierarchy, two distinct populations were identified (Table S3, Figure S1). The O3:K6 serotype isolates belonged to one population, and all other isolates were clustered together in the other population.

**Figure 4:**
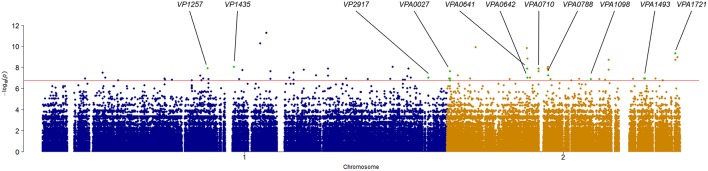
Manhattan plots summarizing the statistical significance of genome-wide associations between core genome SNPs and RTE food source. Missense SNPs are indicated in green plots, and affected genes are shown in the top.

Association analysis was conducted after correction for population structure, and the results revealed 78 core genome SNPs that were significantly associated with RTE food sources (adjusted *P*-value < 0.05, Bonferroni method) (Figure 4). After filtering by separately comparisons, 58 outlier SNPs were finally selected for further analysis (Table S4). Among these, 54 SNPs were located in protein-coding regions, resulting in 9 missense variants and 45 synonymous variants. The missense SNPs affected 8 genes (Table 2), including a glutathione S-transferase, a sodium/glutamate symporter, an outer membrane phospholipase, an ATP-dependent protease, and two regulators (LuxN and LysR).

**Table 2:** Genes affected by filtered missense variants that correlate with RTE foods source.

**GeneName**	**Annotation**	**Missense_variant**	**Synonymous_variant**
VP1257	SanA protein	1	0
VP1435	Sodium/glutamate symporter	1	0
VPA0027	Hypothetical protein	2	0
VPA0641	LysR family transcriptional regulator	1	0
VPA0642	Glutathione S-transferase	1	1
VPA0710	Sensor histidine kinase/response regulator LuxN	1	1
VPA0788	Outer membrane phospholipase A	1	0
VPA1493	ATP-dependent protease LA-like protein	1	0

### Functional Analysis of RTE Food-Related Genes

**Figure 5:**
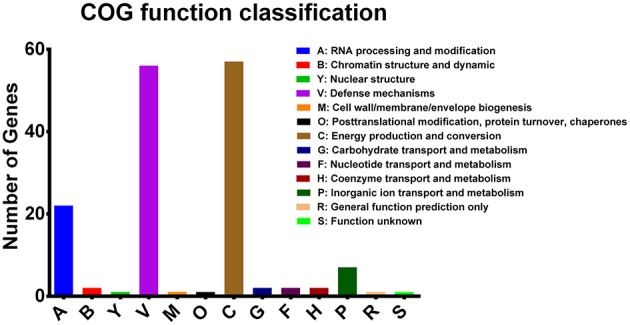
COG functional classifications of *V. parahaemolyticus* RTE food-related genes.

Functional analysis was performed on RTE food-related accessory genes and genes containing missense SNPs according to their COG annotation. Seventy-seven of the Eighty-Seven RTE food-related genes had got the COG annotation, and most of the annotated genes were classified as being involved in defense mechanisms and energy production and conversion (Figure 5). In addition, a proportion of genes were involved in RNA processing and modification and inorganic ion transport and metabolism. We also identified several functional categories that may contribute to the persistence of *V. parahaemolyticus* in RTE foods, including chromatin structure and dynamics, cell wall/membrane/envelope biogenesis, and posttranslational modification/protein turnover/chaperones.

We then analyzed the KEGG pathways of the RTE food-related genes. The biofilm formation pathway was found to be primarily affected by these genes (Table S5, Figure S2). In addition, the quorum sensing pathway was affected, revealing that RTE food-related genes may play an important role in the biofilm formation of *V. parahaemolyticus* in RTE foods. Some of RTE food-related genes were found to be involved in pathways related to drug resistance (e.g., drug metabolism and platinum drug resistance).

### Biofilm Formation of *V. parahaemolyticus* RTE Food Isolates

**Figure 6:**
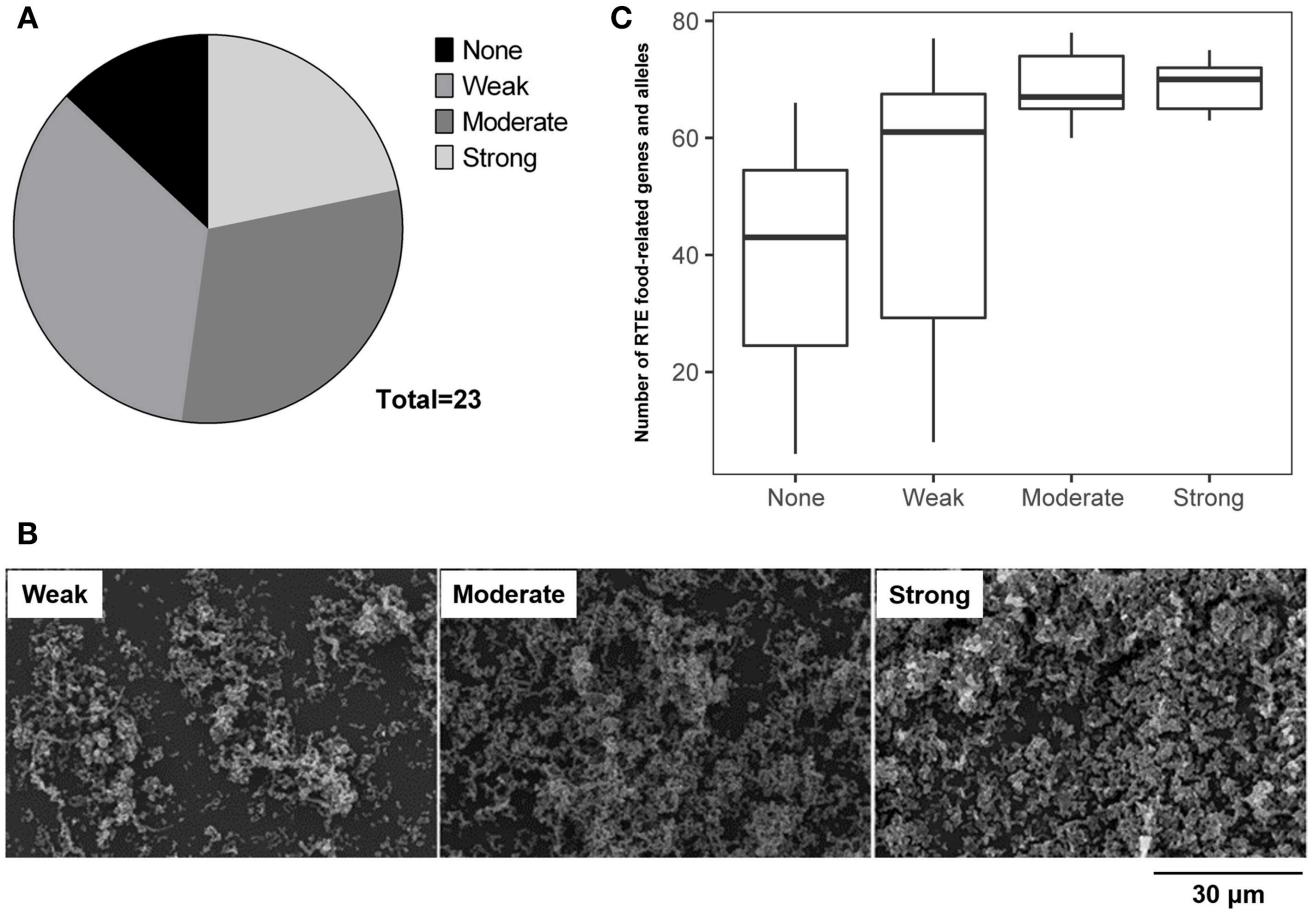
Analysis of the ability of *V. parahaemolyticus* RTE food isolates to form biofilm. **(A)** statistical analysis of the biofilm formation ability of 23 RTE food isolates. **(B)** scanning electron microscope images indicating the formation of weak, moderate, and strong biofilms generated by RTE food isolates. **(C)** number of RTE food-related genes and alleles carried by *V. parahaemolyticus* RTE food isolates possessing various abilities to form biofilm.

The ability of RTE food isolates to form biofilm was assessed using the crystal violet staining method. Twenty-three isolates were tested, among which only three isolates were unable to form biofilm (Figure 6A). Over half of RTE food isolates were able to form a strong or moderate biofilm, while 35% of isolates formed a relatively weak biofilm (Figure 6B). Notably, isolates that were unable to form biofilm or only formed a weak biofilm tended to possess fewer RTE food-related genes and alleles than those isolates that were able to form a strong or moderate biofilm (Figure 6C). Additionally, the ability to form biofilm seemed to have no connection with other features of isolates such as serotypes and virulence factors (Table S6). Taken together, these results indicate that a majority of *V. parahaemolyticus* RTE food isolates possessed biofilm formation ability and that this ability may be closely correlated with the number of RTE food-related genes and alleles present in bacteria.

## Discussion

Bacteria isolated from RTE foods in China are mainly derived from the environment through the contamination of vegetables, incomplete heating, or cross-contamination from the environment (Wu et al., 2015). Our previous study showed that a moderate percentage of Chinese RTE foods are contaminated with *V. parahaemolyticus*, a major food-borne gastroenteritis-causing bacterium (Xie et al., 2016). This bacterium is usually isolated from aquatic products and previous studies were more often focused on these isolates (Letchumanan et al., 2015), while no previous study has explored the pathogenic potential of RTE food isolates. Thus, in this study, we analyzed the genomic features of these isolates for a full understanding of their potential risk. Using next-generation sequencing technology, we obtained the whole genome sequences of 27 *V. parahaemolyticus* RTE food isolates. Subsequent comparative genomics analysis revealed some genomic features specifically found in these isolates in comparison with other *V. parahaemolyticus* isolates.

One of the observed features of RTE food isolates is that they generally possess more protein-coding genes than clinical or environmental isolates (Figure 1). The phenomenon of clinical isolates having fewer genes on average than non-clinical isolates has been observed in both gram-positive and -negative bacteria (Merhej et al., 2013; Weinert et al., 2015) and is hypothesized to result from a reduction in regulatory complexity. However, *V. parahaemolyticus* isolates persisting in RTE foods tended to exhibit increases in genomic complexity instead. This difference may be the result of the diversity of environmental and nutritional stresses facing by different kinds of isolates. To ensure successful invasion and survival in host tissues, pathogenic isolates experience a passive loss of transcriptional regulators but in turn gain more genes encoding toxins, toxin-antitoxin (TA) modules, and proteins involved in DNA replication and repair (Merhej et al., 2013). In contrast, RTE food isolates possess an overrepresentation of genes related to defense mechanisms and energy production and conversion according to their COG annotations (Figure 5), which may due to the specificity of this source. During the processes of RTE food preparation and packaging, bacteria experience multiple kinds of specific stresses, such as heat during pre-cooking, changes in nutritional substrates, drought during transportation, and even disinfectants (Lavieri et al., 2015). The acquisition of additional defense-related genes may therefore be a strategy that allows *V. parahaemolyticus* to tolerate the above stresses so as to persist in RTE foods, and frequent switching among different carriers may require the ability to utilize energy under different energy levels and metabolic yields (Schweinitzer and Josenhans, 2010).

Alternatively, bacteria can also use the strategy of biofilm formation to effectively overcome different environmental stresses (Kubota et al., 2008). Within a biofilm, bacteria are much more resistant to antibiotic treatment (Stewart, 1994; Desai et al., 1998), as well as other environmental stresses (Frank and Koffi, 1990; Kubota et al., 2009). The formation of biofilm is influenced by many factors (Greenberg, 2003), among which quorum sensing (QS) is thought to play a central role (Liaqat et al., 2014). Our analysis demonstrated that some RTE food-related genes are involved in both biofilm formation and QS pathways in *V. parahaemolyticus* (Table S5). In addition, subsequent experiments confirmed that a majority of RTE food isolates possessed the ability to form biofilm, and this ability was positively correlated with the number of RTE food-related genes present (Figure 6). It can thus be seen that if RTE foods are contaminated with these isolates, elimination will be difficult owing to their persistence in the form of biofilms. Thus, the potential threat represented by *V. parahaemolyticus* in RTE foods deserves attention.

Clinical *V. parahaemolyticus* isolates are generally positive for some major virulence factors such as TDH, TRH, VPAIs, and T6SS1. Among which, the post-1995 *V. parahaemolyticus* O3:K6 serotype clone carrying TDH and VPAI-1 to VPAI-7 has disseminated worldwide and is considered to be pandemic (Vuddhakul et al., 2000). Our analysis revealed that only one RTE food isolate (Vp19) expressed TRH, while all other isolates did not produce TDH or TRH, indicating that none of the RTE food isolates belonged to pandemic clone. This finding largely corresponds to the findings of a previous report (Xie et al., 2016). However, Vp19 showed genetic homology to a pre-pandemic O3:K6 isolate (AQ4037). The AQ4037 isolate is positive for the virulence factors *trh*, T3SSβ, and T6SS1 and is pathogenetic in humans (Hazen et al., 2015). The same factors were also present in the Vp19 isolate, suggesting the pathogenic potential of this RTE food isolate. In some other RTE food isolates, we also observed the presence of partial T6SS1 genes, which is mostly associated with pathogenic isolates and may contribute to virulence (Yu et al., 2012; Salomon et al., 2014). Notably, T6SS1 genes in different clinical isolates showed a variation range from 73 to 100% (Ronholm et al., 2016), suggesting that partial T6SS1 genes in RTE food isolates still have the pathogenic possibility. Moreover, some VPAIs were frequently present in various RTE food isolates, reflecting the occurrence of HGT of VPAIs among pathogenic isolates and RTE food isolates. The acquisition of multiple virulence factors by HGT can potentially cause the emergence of new pathogens (Espejo et al., 2017). Additionally, recent studies have reported that some clinical isolates do not possess the known PAIs or only carried part of them (Hazen et al., 2015), and some even showed the absence of the *tdh* and *trh* genes (Jones et al., 2012; Ottaviani et al., 2012; Hazen et al., 2015; Ronholm et al., 2016), as was observed for the VpL83 isolate. Thus, its homologous isolate, Vp43, is probably pathogenic in humans, even though it does not possess all known virulence factors. Together, these findings indicate the non-negligible potential for the pathogenicity of *V. parahaemolyticus* RTE food isolates, and further investigation should be performed to validate them.

In summary, this study illustrates the genomic features of *V. parahaemolyticus* isolated from RTE foods in China. Some of these isolates appear to share similar genetic architecture with clinical isolates and possess some of the known virulence-associated genes, revealing considerable pathogenic potential. Moreover, most RTE food isolates tended to possess genes and alleles that contribute to defense mechanisms and increase biofilm formation in these isolates, and this may promote their persistence on the surfaces of RTE foods. In consideration of the fact that RTE foods do not require further processing before consumption, contamination with pathogens will pose more of a safety risk for consumers. Therefore, the persistence of *V. parahaemolyticus* in RTE foods deserves further assessment in the future, and more efforts should be made to develop effective control strategies.

## Author Contributions

RP and QW conceived and designed the study. TX, JZ, YD, JW, LX, MC, XW, YZ, SZ, and XY performed the samples and data collection. RP, TX, YL, and TL performed the data analysis. RP and QW wrote and finalized the manuscript.

### Conflict of Interest Statement

The authors declare that the research was conducted in the absence of any commercial or financial relationships that could be construed as a potential conflict of interest.
