# Supplementary material for: Comparative Genomic Analysis Reveals the Potential Risk of Vibrio parahaemolyticus Isolated From Ready-To-Eat Foods in China
Source: Front Microbiol. 2019 Feb 7;10:186. doi: 10.3389/fmicb.2019.00186 (PMC6374323; doi:10.3389/fmicb.2019.00186)
Supplement: Supplementary file 1 [file Image_1.pdf]

Supplementary Information of "Comparative Genomic Analysis Reveals the Potential Risk of *Vibrio parahaemolyticus* Isolated from Ready-to-eat Foods in China"

Rui Pang[1], Tengfei Xie[1], Qingping Wu[1*], Yanping Li[1], Tao Lei[1], Jumei Zhang[1], Yu Ding[1], Juan Wang[1], Liang Xue[1], Moutong Chen[1], Xianhu Wei[1], Youxiong Zhang[1], Shuhong Zhang[1] and Xiaojuan Yang[1].

[1]State Key Laboratory of Applied Microbiology Southern China, Guangdong Provincial Key Laboratory of Microbial Culture Collection and Application, Guangdong Open Laboratory of Applied Microbiology, Guangdong Institute of Microbiology, Guangzhou 510070, China

*** Correspondence:**
Qingping Wu
wuqp203@163.com

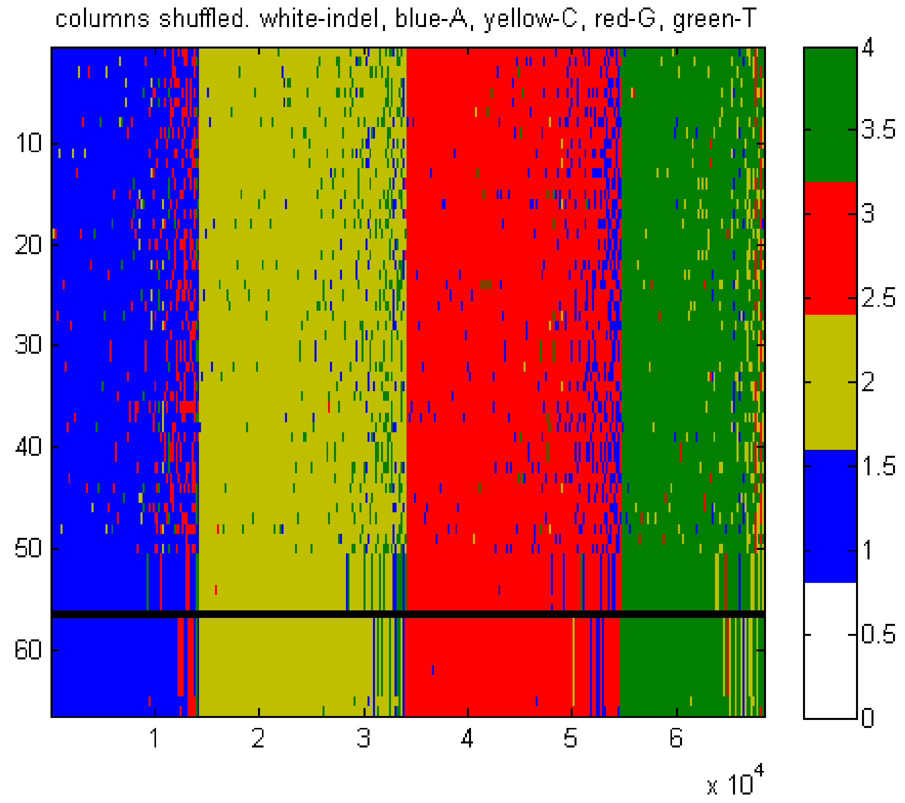

Figure S1. Bayesian Analysis of Population Structure of 66 global isolates of *Vibrio parahaemolyticus*. Each row in the figure is a strain and each column is a SNP, and horizontal black lines are drawn to separate the two clusters. The colour pattern of these SNPs within the black lines is consistent with them being assigned to the same BAPS group.

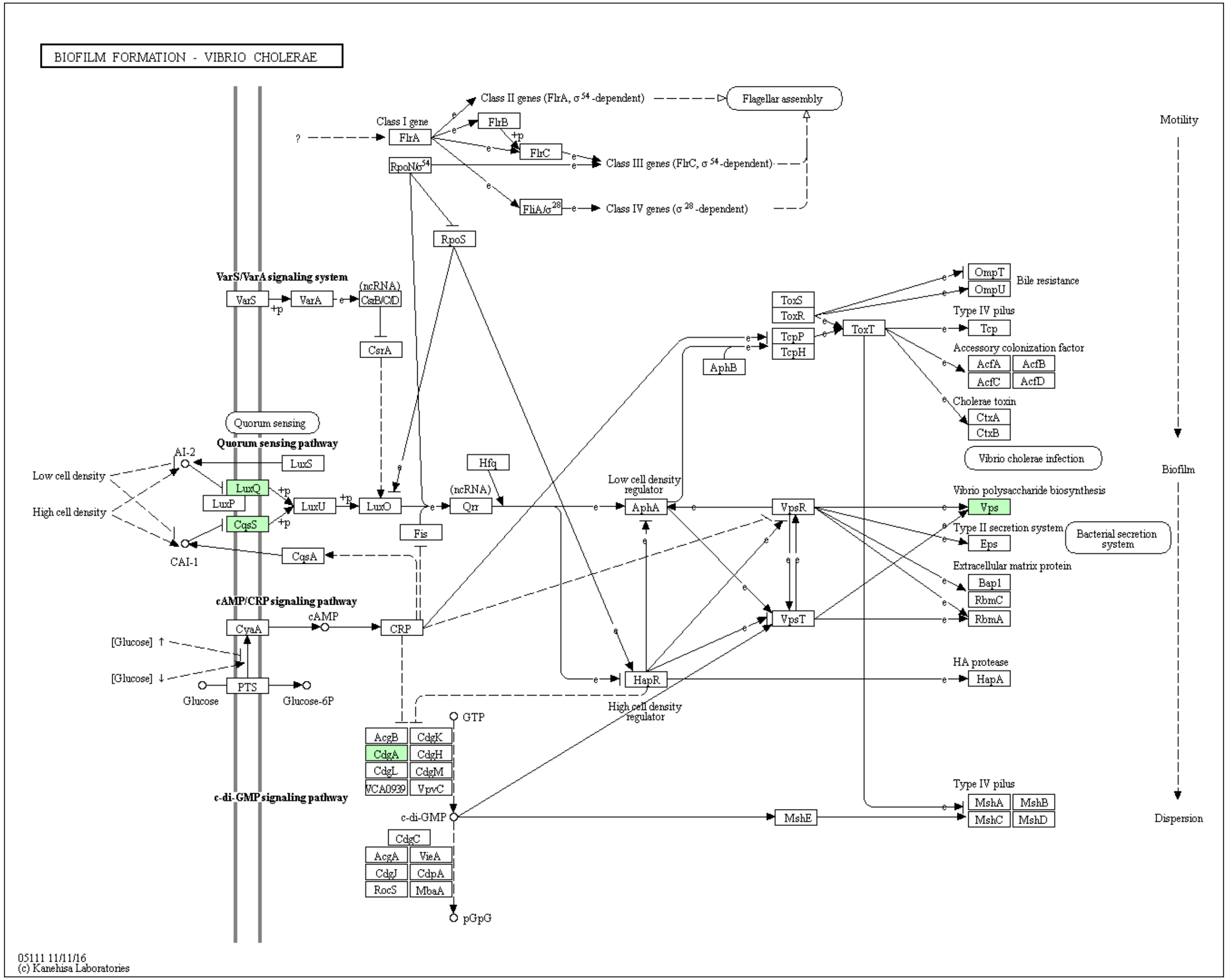
BIOFILM FORMATION - VIBRIO CHOLERAE
Class II genes (FlrA, σ 54 -dependent)
Flagellar assembly
Class I gene
FlrA
FlrB
+p
FlrC
RpoN/σ54
Class III genes (FlrC, σ 54 -dependent)
FliA/σ28
Class IV genes (σ 28 -dependent)
?
RpoS
Motility
VarS/VarA signaling system
VarS
+p
VarA
(ncRNA)
CsrB/C/D
CsrA
ToxS
ToxR
TcpP
TcpH
AphB
ToxT
OmpT
OmpU
Bile resistance
Type IV pilus
Tcp
Accessory colonization factor
AcfA
AcfB
AcfC
AcfD
Cholerae toxin
CtxA
CtxB
Vibrio cholerae infection
Quorum sensing
Quorum sensing pathway
AI-2
LuxS
Low cell density
High cell density
LuxQ
LuxP
+p
LuxU
LuxO
CqsS
CqsA
CAI-1
Hfq
(ncRNA)
Qrr
Fis
Low cell density regulator
AphA
VpsR
VpsT
Biofilm
Vibrio polysaccharide biosynthesis
Vps
Type II secretion system
Eps
Bacterial secretion system
Extracellular matrix protein
Bap1
RbmC
RbmA
cAMP/CRP signaling pathway
CyaA
cAMP
CRP
[Glucose] ↑
[Glucose] ↓
PTS
Glucose
Glucose-6P
HapR
High cell density regulator
HA protease
HapA
GTP
AcgB
CdgK
CdgA
CdgH
CdgL
CdgM
VCA0939
VpvC
c-di-GMP signaling pathway
c-di-GMP
MshE
Type IV pilus
MshA
MshB
MshC
MshD
Dispersion
CdgC
AcgA
VieA
CdgJ
CdpA
RocS
MbaA
pGpG
05111 11/11/16
(c) Kanehisa Laboratories

Figure S2. Pathway of biofilm formation (Ko05111). Green indicates the RTE food-related genes. LuxQ: group_2245 (K10909); cqsS: VPA0710 (K10916); exoP: group_261 (K16554); vpsM: group_6406 (K20920); cdgA: group_3579 (K20956).
